# Effects of Pterostilbene on Cardiovascular Health and Disease

**DOI:** 10.3390/cimb46090569

**Published:** 2024-08-30

**Authors:** Rui Tian, Lingchao Miao, Wai-San Cheang

**Affiliations:** State Key Laboratory of Quality Research in Chinese Medicine, Institute of Chinese Medical Sciences, University of Macau, Macau SAR, China; mc36204@um.edu.mo

**Keywords:** pterostilbene, cardiovascular disease, diabetes, antioxidant, bioavailability

## Abstract

Pterostilbene is a phenolic compound commonly found in blueberries, peanuts, grapes, and other plants. It is a dimethoxy derivative of resveratrol. In recent years, it has gained significant attention due to its remarkable anti-inflammatory and antioxidant effects. In addition, its high bioavailability and low toxicity in many species has contributed to its promising research prospects. Cardiovascular disease is closely related to pathological processes such as inflammation and oxidative stress, which aligns well with the treatment applications of pterostilbene. As a result, numerous studies have investigated the effects of pterostilbene on cardiovascular health and disease. This paper summarizes the current research on pterostilbene, with a specific focus on its potential therapeutic role in treating cardiovascular disease.

## 1. Introduction

**Figure 1:**
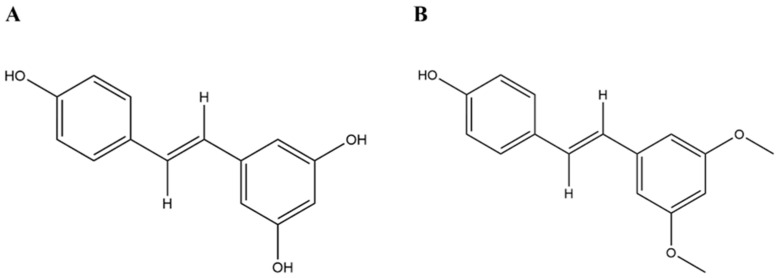
Structures of resveratrol (**A**) and pterostilbene (**B**).

Pterostilbene, a trans-stilbene compound and a dimethylated derivative of resveratrol, belongs to the polyphenolic group of compounds (Figure 1). In recent years, the research on pterostilbene has attracted tremendous attention, focusing on various aspects including its source, chemical structure, biological activity, and action mechanisms. Possessing a variety of pharmacological properties, pterostilbene has bioactive potential in anti-inflammatory, anti-cancer, anti-cardiovascular disease, and anti-aging treatments. While pterostilbene shares similarities with resveratrol and demonstrates partially overlapping pharmacological functions, its unique dimethyl structure allows for distinct biochemical and pharmacological properties compared to resveratrol.

Cardiovascular disease has emerged as a leading cause of morbidity and mortality worldwide. Featuring chronic vascular inflammation, cardiovascular diseases have a complex pathological process, including hypertension and atherosclerosis. Vascular inflammation is closely related to vascular endothelial dysfunction and oxidative stress, which are important drug targets for the treatment of cardiovascular diseases. This review aims to explore the pharmacological effects, related targets, and mechanisms of pterostilbene in the cardiovascular system, thereby providing insights into the future directions of pterostilbene research in the cardiovascular field.

## 2. Sources of Pterostilbene

### 2.1. Natural Sources of Pterostilbene

Pterostilbene is present in various parts of plants, including the leaves, heartwood, and fruit (Table 1). It was first isolated from the heartwood of red sandalwood in 1940. Besides red sandalwood, pterostilbene can be found in many other plant species, with notable concentrations in grape, blueberry, and Vaccinium (*V. ashei*) varieties. It was also found in *Pterocarpus marsupium*, a Kino tree in India, and *Guibourtia tessmanii* in Africa [1]. The existence of pterostilbene in diverse plants is related to its potential plant antitoxic function. As a secondary metabolite in plants, pterostilbene plays a practical role in the plant’s defense against adverse environmental challenges, such as microbial and fungal infections, ultraviolet exposure, high temperatures, and heavy metal pollution. Pezet’s team has found that the content of pterostilbene in grape peels infected with fungi is significantly higher than that in uninfected grape peels [2]. This suggests that pterostilbene may contribute to the detoxification response of plants to unfavorable environmental conditions.

### 2.2. Synthesis Methods of Pterostilbene

There are many natural sources of pterostilbene. As a secondary metabolite in plants, pterostilbene can be extracted and isolated from various plant species. However, the amount of pterostilbene obtained from pure natural sources is often limited, which has prompted significant interest in developing chemical and biosynthesis methods to obtain this substance in larger quantities.

From a structural perspective, both resveratrol and pterostilbene are natural products sharing a stilbene backbone. Resveratrol is a trihydroxy derivative of trans-stilbene, while pterostilbene is a dimethoxy derivative of resveratrol. In existing biosynthesis methods of pterostilbene, resveratrol is often used as a substrate, and the synthesis of pterostilbene is catalyzed by O-methyltransferase, which modifies the A ring of resveratrol [9]. Rimando’s team has successfully synthesized and characterized pterostilbene by using *O*-methyltransferase from *Sorghum bicolor* and resveratrol as a substrate [10]. Stilbene synthase (STS) is another crucial enzyme involved in pterostilbene synthesis. STS catalyzes the synthesis of the parent stilbene structure using *p*-coumaroyl-CoA as a starting unit, followed by modifications to the benzene ring to produce various stilbene derivatives, including pterostilbene. Biosynthesis methods generally offer high yields and relatively lower production costs, making them significant in pterostilbene synthesis.

The starting elements for the synthesis of pterostilbene typically include derivatives containing benzene rings, such as 3,5-dihydroxyacetophenone, 3,5-disubstituted benzyl bromide, 5-dimethoxybenzyl sulfone derivative, and 4-acetoxy benzaldehyde. These derivatives are first synthesized to obtain the parent structure of stilbene, which is then subjected to subsequent reactions to yield the final pterostilbene product. Biosynthesis methods are generally more economical and environmentally friendly compared to chemical synthesis methods.

## 3. Biological Activities

Resveratrol and its dimethoxy analog pterostilbene share similarities in their pharmacological functions. As polyphenolic derivatives, both compounds have anti-inflammatory and antioxidant effects. The chemical structural differences between pterostilbene and resveratrol confer certain advantages to pterostilbene. One notable distinction is the better lipid solubility of pterostilbene compared to resveratrol, resulting in the better bioavailability and improved pharmacokinetic profile of pterostilbene.

Endothelial function plays a critical role in cardiovascular health as it directly affects vascular homeostasis and the normal physiological function of the cardiovascular system. Multiple vascular functions such as anti-inflammatory responses, anticoagulant properties, and vasodilation are all regulated by the endothelium [11]. Moreover, vascular inflammation, oxidative stress, and endothelial dysfunction are closely associated with diseases such as diabetes, hypertension, and obesity, contributing to cardiovascular complications. Therefore, preventing, controlling, and alleviating vascular inflammation and oxidative stress are considered important resolutions for treating cardiovascular diseases.

**Figure 2:**
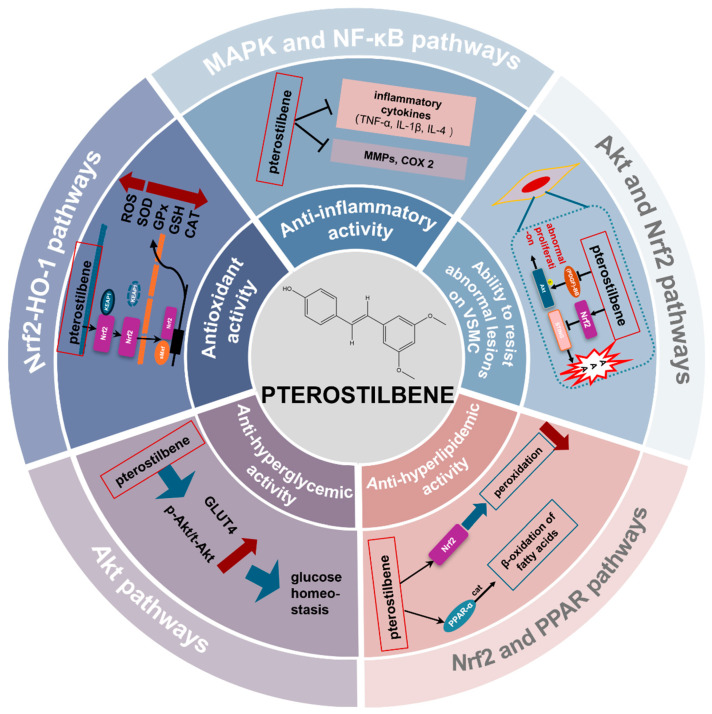
Therapeutic properties of pterostilbene in cardiovascular diseases and underlying mechanisms.

Extensive evidence has supported that pterostilbene has a variety of pharmacological effects, such as anti-oxidative, anti-inflammatory, and anti-hypoglycemic properties, implying its potential benefits in the prevention and management of cardiovascular diseases. Here, we outlined the current research mechanisms of pterostilbene in treating cardiovascular diseases (Figure 2).

### 3.1. Antioxidant Activity

Pterostilbene has been extensively studied for its remarkable antioxidant and anti-inflammatory bioactivities. Pterostilbene could significantly enhance antioxidant capacity during diabetes and improve tissue resilience against oxidative stress [12,13]. To identify the anti-oxidative capacity of pterostilbene, the common immune cell model, RAW264.7 cells induced by advanced glycation end products (AGEs) to mimic the oxidative stress of diabetes, was chosen by Yu’s research team. Pterostilbene significantly down-regulates nicotinamide adenine dinucleotide phosphate (NADPH) oxidase (NOX), which is the key enzyme family that induces the release of reactive oxygen species (ROS) in AGE-induced RAW264.7 cells [14]. Apart from this, the damage of hepatocytes and kidney cells is alleviated by pterostilbene treatment as it increases the expression levels of catalase (CAT), glutathione (GSH), superoxide dismutase (SOD), and other antioxidants in diabetic rats [15]. Likewise, two-month administration of blueberry extract containing pterostilbene increases the biological activities of SOD and glutathione peroxidase (GPx) and decreases the level of a diabetes index, the HbA1c enzyme, in diabetic patients [16].

Oxidative stress, generally induced by ROS, can lead to detrimental effects on the normal function of vascular endothelial cells. As an important regulator of oxidative stress, nuclear factor erythroid 2-related factor (Nrf2) has been shown to inhibit oxidative stress-induced damage in endothelial cells in obesity models, along with activating the Nrf2/heme oxygenase-1 (HO-1) signaling pathway, which has been identified as a potential mechanism for alleviating hypertension, atherosclerosis, and other cardiovascular diseases [17,18]. In a streptozotocin (STZ)-induced diabetic model, the administration of pterostilbene leads to the significant elevation of Nrf2 levels. This activation of the Nrf2 pathway restores the activities of redox-related enzymes in the liver, including SOD, CAT, and GPx [19]. Compared with Western diet-fed ApoE^−/−^ mice, pterostilbene treatment reverses the elevation of malondialdehyde (MDA) and H_2_O_2_, demonstrating its outstanding anti-oxidative activity [20]. In conclusion, pterostilbene works as an activator of Nrf2 to exhibit anti-oxidative effects.

### 3.2. Anti-Inflammatory Activity

In both in vitro and in vivo experiments, pterostilbene also shows a satisfying anti-inflammatory effect. Pro-inflammatory mediators including pro-inflammatory cytokines (tumor necrosis factor (TNF)-α, interleukin (IL)-1β, and IL-4), matrix metalloproteinases (MMPs), and cyclooxygenase (COX)-2 are all suppressed by pterostilbene treatment. Additionally, the anti-inflammatory action of pterostilbene has been proved to be associated with modulating mitogen-activated protein kinase (MAPK) and nuclear factor kappa B (NF-κB) pathways [21,22]. The activation of endoplasmic reticulum stress will lead to subsequent endothelial cell inflammation, which can be alleviated by treatment with pterostilbene. Liu’s team has proved that pterostilbene can successfully reverse the elevation of related pro-inflammatory cytokines (IL-8, monocyte chemoattractant protein (MCP)-1, and E-selectin) induced by TNF-α activation in human umbilical vein endothelial cells (HUVECs), adequately demonstrating the ability of pterostilbene to relieve endothelial inflammation [23].

Atherosclerosis is a well-known chronic cardiovascular disease characterized by the accumulation of plaque in the arterial walls. Its pathogenesis involves a variety of factors, including hyperlipidemia, high cholesterol, hypertension, and diabetes. These risk factors can lead to inflammation and oxidative stress within blood vessels, which further cause damage to the arteries and promote the development of atherosclerosis. Pterostilbene can exert anti-atherosclerotic activity through effectively restricting the secretion of pro-inflammatory cytokines, such as interferon (IFN)-γ, IL-6, and TNF-α, modulating the release of CAT in vascular smooth muscle cells (VSMCs), and regulating VSMC function [20]. Normally, apolipoprotein E knockout (ApoE^−/−^) mice are chosen to set up an atherosclerotic animal model by being fed a Western diet. After 16-week intragastrical administration, pterostilbene could significantly curb the growth and development of thoracic and abdominal aortic plaques in ApoE^−/−^ mice by improving lipid metabolism and inhibiting oxidative stress and inflammation in vivo [20]. Experiments conducted by Xiong’s lab on the Sprague-Dawley (SD) rat model also verified the anti-atherosclerosis effect of pterostilbene. In atherosclerotic rats fed with a 2.5% cholesterol Western diet for 12 weeks, treatment with pterostilbene in the last 4 weeks reversed aortic inflammation and oxidative stress in atherosclerotic rats by modulating Nrf2-mediated TLR-4/MyD88/NF-κB signaling pathways, which has been further verified by in vitro experiments using endothelial cells [24].

### 3.3. Anti-Hyperlipidemic Ability

One of the major characteristics of both cardiovascular disease and diabetes is dyslipidemia. Nrf2 is involved in regulating lipid metabolism. Pterostilbene has been demonstrated to reduce lipid peroxidation by regulating the expression of Nrf2, exhibiting anti-peroxidation and anti-hyperlipidemic effects [19]. Peroxisome proliferator-activated receptors (PPARs) participate in the regulation of lipid, lipoprotein, and glucose homeostasis, and they impact cell proliferation, differentiation, and apoptosis in many tissues. PPAR-α is a specific target related to dyslipidemia due to its participation in the metabolism of fatty acids and lipids. PPAR-α catalyzes the β-oxidation of fatty acids by activating genes involved in fatty acid oxidation. Moreover, PPAR-α is the target of lipid-regulating drugs known as fibrates. Pterostilbene acts as a potent PPAR-α agonist [25,26]. Luciferase activity was induced by Rimando using H4IIEC3 cells (a rat hepatoma cell line) transfected with a peroxisome proliferator response element, AB (a rat fatty acyl CoA β-oxidase response element), and a luciferase gene reporter to determine whether pterostilbene is capable of activating PPAR-α. They found that pterostilbene effectively activates PPAR-α, leading to 8- to 14-fold increases in luciferase activity compared to the model group. In male golden Syrian hamsters, pterostilbene exerts significant effects on glucose and lipid metabolism [1]. Compared to the high-fat diet group, the administration of pterostilbene could effectively reduce the plasma low-density lipoprotein (LDL) cholesterol levels of hamsters by 29% and increase the plasma high-density lipoprotein (HDL) cholesterol levels by almost 7%, as well as decreasing blood glucose levels by about 14% via activating PPAR-α [27]. In addition to PPAR-α, PPAR-γ and the other PPAR family members also engage in the differentiation of adipose and assist with lipid storage at the same time [25]. Given the significant involvement of PPAR-γ in the generation of adipose, PPAR-γ is naturally deemed one of the master mediators of lipid metabolism [26]. Pterostilbene has been proved to diminish adipogenesis by effectively suppressing the expression of PPAR-γ in human mesenchymal stromal cells (hMSCs) [28].

Oxidized low-density lipoprotein (ox-LDL) is a key contributor to the development of atherosclerosis. Normal LDL in blood vessels can undergo oxidation and modification, forming ox-LDL. The increase and accumulation of ox-LDL leads to a series of vascular lesions. Endothelial injury triggers monocyte chemotaxis and abnormal proliferation of smooth muscle cells, leading to the formation of foam cells and atherosclerotic plaques. Pterostilbene is capable of resisting atherosclerosis induced by ox-LDL. This beneficial activity of pterostilbene is mainly achieved by inhibiting the harmful apoptosis process and stimulating the beneficial autophagy process in vascular endothelial cells. The beneficial autophagy process induced by pterostilbene promotes the clearance of ox-LDL-derived harmful proteins including p62 and LC3-II deposited in endothelial cells [29,30].

Six-week administration of pterostilbene (40 mg/kg) significantly elevates anti-oxidative enzyme activities in liver and kidney tissues of streptozotocin–nicotinamide-induced diabetic rats. This demonstrates that pterostilbene improves the anti-oxidative capacity of diabetic rats [31]. Nowadays, a product named PTeroPure containing pterostilbene has been patented, created by ChromaDex in the United States with the aim of lowering cholesterol and preventing age-related cognitive disorders.

### 3.4. Anti-Hyperglycemic Ability

Blood glucose and lipid homeostasis are two key factors that influence the progression of cardiovascular diseases. Currently, the positive effect of pterostilbene on hypoglycemia has been proved by several pre-clinical studies and clinical trials. Based on the successfully established obese and insulin-resistant Wistar rat model, the experimental group was administered with pterostilbene at low (15 mg/kg/day) and high (30 mg/kg/day) concentrations by S. Gómez-Zorita’s team. They found that pterostilbene activates protein kinase B (Akt) and significantly up-regulates the ratio of phosphorylated Akt/total Akt in the skeletal muscle tissues of the high-dose group. Glucose transporter type 4 (GLUT4) is an important protein that mediates glucose uptake and plays an important role in maintaining glucose homeostasis. Upon stimulation, GLUT4 is transferred from vesicles to the plasma membrane. Akt activates GLUT4 translocation in insulin cascade signaling, which provides insight into the close relationship between Akt and GLUT4 in the maintenance of glucose homeostasis [32]. According to Gómez-Zorita’s results, the expression levels of GLUT4 in rats of both the low- and high-dose pterostilbene-treated groups are all significantly up-regulated. These results suggest that the anti-hyperglycemic effect of pterostilbene mainly relies on the activation of the Akt signaling pathway [33]. In streptozotocin- and nicotinamide-induced diabetic male albino Wistar rats, pterostilbene at different doses of 10, 20, and 40 mg/kg was used to treat diabetic rats, and then, the fasting blood glucose levels of rats were measured at 2, 4, and 6 weeks. The results showed that only high-dose (40 mg/kg) pterostilbene treatment could significantly reduce blood glucose levels and increase plasma insulin and total hemoglobin levels, accompanied by a significant decrease in glycosylated hemoglobin levels, verifying the excellent anti-hypoglycemic capacity of pterostilbene, which reflects its potential to regulate glucose homeostasis [15]. Moreover, the PI3K/Akt signaling pathway exhibits a non-negligible function in controlling glucose homeostasis, which is improved by pterostilbene in STZ-induced diabetic SD rats, and is involved in the anti-hyperglycemia activity of pterostilbene [34].

### 3.5. Ability to Resist Abnormal Lesions of VSMCs

Besides endothelial cells, some other elements like VSMCs as major cellular components of the arterial wall are involved in maintaining cardiovascular homeostasis and therefore cannot be ignored. Once an artery has suffered an injury, a variety of cells, such as vascular endothelial cells, VSMCs, and macrophages, will immediately release growth factor platelet-derived growth factor (PDGF)-BB, which will further promote the proliferation of fibroblasts and VSMCs, leading to vascular stenosis and eventually hypertension and atherosclerosis [35]. It has been reported that pterostilbene apparently inhibits the abnormal proliferation of VSMCs stimulated by PDGF-BB. The IC_50_ values of pterostilbene on VSMCs and DNA synthesis were 1.53 ± 0.04 μmol/L and 1.08 ± 0.02 μmol/L, respectively. Pterostilbene (5 μmol/L) exhibited complete suppression of the synthesis of VSMCs and DNA through inhibiting the Akt-dependent pathway. According to further investigations, the relationship between (PDGF)-BB and Akt has been well noted, and Park’s team has proved that pterostilbene could lock down (PDGF)-BB’s ability to induce phosphorylation of Akt by decreasing the expression of (PDGF)-BB [36].

One of the most critical cardiovascular diseases is abdominal aortic aneurysm (AAA), which includes multiple pathological characteristics like the functional degeneration of the elastic lamina within the lamellae aorta; an inflammatory reaction in vascular-related organs; the uncontrolled over-release of ROS; and the overactivation of MMPs [37,38]. The Kelch-like ECH-associated protein 1 (KEAP1)-Nrf2–simulator of interferon genes (STING) signaling pathway has been verified to assist pterostilbene in preventing the formation of AAA in VSMCs, based on Zou’s experimental results [38]. Pterostilbene can combine with KEAP1 competitively, and once it succeeds, it will subsequently up-regulate Nrf2 and the related downstream genes. The activation of Nrf2 is responsible for the inhibition of the STING pathway and its related inflammatory cascades in VSMCs [38].

### 3.6. Ability to Protect against Myocardial Infarction

Myocardial infarction (MI) results in high mortality and morbidity rates all over the world and is caused by multiple risk factors, including apoptosis of cardiomyocytes, deadly infiltration of inflammatory cells, rapid elevation of reactive species, free radicals, and irrevocable DNA damage [39,40,41]. As an acute cardiovascular event, myocardial infarction is universally recognized as rapid myocardial cell death followed by ischemia for a prolonged period, which affects the balance of blood supply and disrupts the demands of myocardial cells [39,42]. Regarding classification of MI, there are five types of MI based on the pathogenesis and circumstances of the illness (Table 2).

Reactive species, including ROS and reactive nitrogen species (RNS), generated from myocardial ischemia will aggravate the release of pro-inflammatory cytokines and chemokines to induce rehabilitation in the infarcted region and cardiac remodeling [41]. The brilliant antioxidant capacity of pterostilbene was found to protect against acute MI in cardiac and pulmonary tissues in Wistar rats [45]. Pterostilbene reversed the reduction in antioxidant enzymes in the right ventricle (RV) after infarction and significantly boosted glutathione, SOD, and CAT [45]. Mastering the timing of MI treatment to conduct reperfusion of the myocardium has become one of the universal medical methods to alleviate MI [46]. Unfortunately, reperfusion cuts both ways in that it can cause a series of complications, making the cardiac situation worse. The above symptoms are comprehensively defined as myocardial ischemia/reperfusion (MI/R) injury [47]. Yu’s team demonstrated that pterostilbene treatment improved cardiac function and decreased myocardial injury and apoptosis after MI/R by performing experiments on adult male SD rats. Given the close relationship between apoptosis and mitogen-activated protein kinase (MAPK) cascades, previous work has proved that p38 MAPK activation participates in neutrophil infiltration, which was apparently suppressed by pterostilbene administration in myocardial ischemia–reperfusion model rats [48,49]. Moreover, oxidative/nitrative stress induced by MI/R injury were strongly suppressed by pterostilbene treatment through the inhibition of oxidative stress [50]. Regarding other reactions which are induced by MI/R in rat heart tissues, Lv et al. investigated multiple other therapeutic roles of pterostilbene. They found that pterostilbene not only inhibits neutrophil accumulation but also deters the release of cardiovascular harmful factors and enzymes, specifically myocardial myeloperoxidase, serum creatinine kinase, lactate dehydrogenase, and TNF-α, which comprehensively proves the cardio-protective and anti-inflammatory bioactivities of pterostilbene in cardiovascular systems [51].

### 3.7. Ability to Protect against Stroke

Stroke ranks among the top ten reasons for death worldwide, according to the World Health Organization (WHO 2019), and its incidence has relentlessly increased, especially in low- and middle-income countries [52]. It has been reported that about 50% of survivors are left with irreversible sequalae, resulting in heavy financial and economic burdens on both their family and society [53,54]. The reduction or complete block of blood supply to brain tissues and insufficient oxygen and nutrients subsequently lead to stroke [52,54]. Among stroke patients, ~87% are ischemic, ~10% suffer from intracerebral illnesses, and the rest belong to subarachnoid hemorrhages [55]. The pathological adverse effects of stroke include a high production of free radicals, increased inflammatory reactions, an elevation of intracellular Ca^2+^ levels, and cell death through apoptosis and necrosis [56]. Khan’s team illustrated the therapeutic effects of pterostilbene against MI/R, brain stroke, and associated behavioral symptoms in SD rats [56]. The underlying mechanisms are attributed to the antioxidant activity of pterostilbene and its ability to decrease DNA damage, scavenge free radicals, and modulate neurodegeneration-induced brain damage [56].

Platelets might contribute to both myocardial infarction and cerebral stroke, playing a critical role in cardiovascular inflammation and thrombosis. Once platelets are activated in the rupture procedure of atherosclerotic plaques, the likelihood increases for more fatal cardiovascular diseases, such as atherosclerosis, myocardial infarction, and ischemic stroke [57,58]. The NF-κB signaling pathway is strongly associated with the activation of platelets and inflammation. Relative to BAY11-7082, the IκB kinase (IKK) inhibitor, pterostilbene more effectively reduced platelet activation by inhibiting the NF-κB pathway [57].

The various biological activities of pterostilbene were summarized in Table 3.

## 4. Discussion and Conclusions

With its traditional stilbene phenolic structure, pterostilbene is similar to a series of other compounds with the same parent structure. As a traditional dietary antioxidant, pterostilbene has an excellent anti-oxidative capacity. Moreover, anti-inflammatory activity is another research hotspot of pterostilbene. According to the existing literature, pterostilbene can exert excellent anti-inflammatory and anti-oxidative abilities, which provides a solid molecular foundation for further clinical trials of pterostilbene. Moreover, pterostilbene exhibits excellent anti-inflammatory potential in various fields, such as obesity, atherosclerosis, and certain cancers. Multiple molecular signal pathways are associated with the anti-inflammatory, anti-oxidative, anti-hyperglycemic, and anti-hyperlipidemic activities of pterostilbene and can be seen as the potential mechanisms for treating various diseases, including the NF-κB pathway, the PPAR pathway, the Nrf2 pathway, the AMPK pathway, the MAPK pathway, and the Akt/GLUT4 signaling pathway, as summarized in Figure 2. Apart from those, pterostilbene (1 μmol/L) could inhibit atherosclerosis progression by preventing cell apoptosis stimulated by ox-LDL [30]. The Nrf2 signaling pathway is the main transportation hub for maintaining blood glucose homeostasis and suppressing vascular smooth muscle lesions, and it is also the critical regulator of peroxidation of lipids and capable of inhibiting STING and subsequent AAA.

Cardiovascular diseases consist of various symptoms, affected by multiple factors including physiological, environmental, and lifestyle factors. Each CVD has its own unique definition and pathological characteristics, while redox imbalance and inflammatory responses both play important roles in CVD progression. Most of pterostilbene’s effects on CVDs are closely related to its antioxidant and anti-inflammatory bioactivities.

The development of CVDs is associated with a variety of factors, with cross-influences between cells, cytokines, and signaling pathways finally resulting in obesity, hypertension, hyperlipidemia, and diabetes, all of which further affect the normal function of the cardiovascular system. The beneficial effects of pterostilbene on cardiovascular disease have contributed to more potential applications. However, this review only provides a brief overview of the bioactivities of pterostilbene in the field of cardiovascular disease. Pterostilbene also has benefits in the application of treating other diseases. For instance, pterostilbene at a dose of 60 μg/mL could completely inhibit the germination of conidia of Botrytis cinerea, showing its excellent antimicrobial capacity [59]. The anti-inflammatory and antioxidant properties of pterostilbene also make it play an important role in the treatment of Alzheimer’s disease and vascular dementia [60]. Based on the various research works on pterostilbene, we believe that the application on pterostilbene is promising in the field of cardiovascular disease.

## Figures and Tables

**Table 1:** Natural sources and content of pterostilbene.

Parts of Plants	Source	Content of Pterostilbene	Reference
Heartwood	Red sandalwood (*Pterocarpus santalinus*)	Pterostilbene was first isolated from the heartwood of red sandalwood in 1940	[3]
Skin	Grapes (non-UV-induced berries of Chardonnay variety)	~4.7 μg/g fresh samples	[4]
Fruit	Rabbit-eye blueberry (*Vaccinium ashei*)	99–151 ng/g dry samples	[5]
Fruit	Deerberry (*Vaccinium stamineum*)	520 ng/g dry samples	[5]
Fruit	Blueberry	9.9–15.1 μg/kg fresh samples	[6]
Wood	*Pterocarpus marsupium*	No specific information	[7]
stem bark	*Guibourtia tessmanii*	40 mg/kg dry samples	[8]

**Table 2:** Five common classifications of myocardial infarction (MI).

Types	Classification	Pathogenesis
Type 1	Spontaneous MI	Accompanied by atherosclerosis, intraluminal thrombus occurs in coronary arteries, subsequently slowing down blood flow velocity and blocking the vessels, which finally leads to myocyte necrosis [43,44].
Type 2	MI secondary to an ischemic imbalance	There is an imbalance between increasing oxygen demand precipitated by conditions like hypertension and a decreasing supply of oxygen due to coronary artery spasm, arrhythmia, hypotension, etc. [44].
Type 3	MI caused by sudden unexpected cardiac death	Sudden cardiac death (SCD) arises due to acute myocardial ischemia [43].
Type 4 and 5	MI related to revascularization procedures	Whether from percutaneous coronary intervention (PCI) or coronary artery bypass grafting (CABG), patients with periprocedural myocardial injury or infarction may suffer from MI [42].

**Table 3:** Summary of biological activities of pterostilbene.

Diseases	Signaling Pathways	Models	Dose	Reference
Inflammation	RAGE/MAPK/NF-κB pathways	RAW264.7 cells induced by AGEs to create oxidative stress and inflammation	10 μM	[14]
Inflammation	Endoplasmic reticulum stress (ERS) pathways	HUVECs induced by TNF-α	1 μM	[23]
Atherosclerosis	CAT-PTEN pathways	HFD-fed ApoE^−/−^ mice	30 mg/kg/d i.g.	[20]
Atherosclerosis	Nrf2-mediated TLR-4/MyD88/NF-κB pathways	SD rats fed with a 2.5% cholesterol diet	10 mg/kg/day orally	[24]
Dyslipidemia	PPAR-α pathways	HFD-fed male golden Syrian hamsters	25 mg/kg/day orally	[27]
Dyslipidemia	PPAR-γ pathways	Human mesenchymal stromal cell (hMSC) adipogenic differentiation	5 μM	[28]
Apoptosis	Lectin-like ox-LDL receptor1 (LOX-1)-related pathways	HUVECs induced by ox-LDL	1 mM	[29]
Diabetes	Nrf2 pathway	Streptozotocin-induced diabetic Swiss albino mice	5 mg/kg i.p. 10 mg/kg i.p.	[19]
Diabetes	/	Streptozotocin–nicotinamide-induced diabetic male albino Wistar rats	40 mg/kg/day orally	[31]
Diabetes	Akt pathways	Streptozotocin- and nicotinamide-induced diabetic male albino Wistar rats	15 mg/kg/day orally (low) 30 mg/kg/day orally (high)	[33]
Diabetes	Nrf2 pathways	Streptozotocin-induced diabetic Swiss albino mice	10 mg/kg i.p.	[34]
Lesions of VSMCs	Akt pathways	Platelet-derived growth factor (PDGF)-BB-induced abnormal proliferation of VSMCs	1 μM (low) 3 μM (middle) 5 μM (high)	[36]
Abdominal aortic aneurysm (AAA)	KEAP1-Nrf2-STING pathways	Angiotensin II (AngII)-infused ApoE^−/−^ mice	15 mg/kg/day orally (low) 30 mg/kg/day orally (high)	[38]
Myocardial infarction (MI)	Nrf2 pathways	Male Wistar rats with ligation and reperfusion-induced myocardial infarction	100 mg/kg/day orally	[45]
Myocardial ischemia/reperfusion (MI/R) injury	P38 MAPK and ROS-related pathways	Male Sprague-Dawley rats with MI/R surgery around the left anterior descending coronary artery (LAD)	10 mg/kg i.v. 10 min before reperfusion	[50]
Myocardial ischemia/reperfusion (MI/R) injury	TNF-α and cGMP-related pathways	Male Sprague-Dawley rats with MI/R surgery on left heart	10 μM i.v. 10 min before reperfusion	[51]
MI/R and stroke	/	Male Sprague-Dawley rats with MI/R surgery on bilateral carotid artery	200 mg/kg orally (low) 400 mg/kg orally (high)	[56]
Inflammation and thrombosis	NF-κB pathways	Platelets activated by collagen	3 μM (low) 7 μM (high)	[57]
